# Novel machine learning-based approach to identify viral biomarkers of human respiratory emissions from oral and nasal metagenomes

**DOI:** 10.1128/msphere.00113-26

**Published:** 2026-04-13

**Authors:** Kathryn Langenfeld, Peter Arts, Abigail Monahan, Allyson Criswell, Krista R. Wigginton, Melissa B. Duhaime

**Affiliations:** 1 Department of Ecology and Evolutionary Biology, University of Michigan 118714, Ann Arbor, Michigan, USA; 2 Department of Civil and Environmental Engineering, University of Michigan 542596, Ann Arbor, Michigan, USA; Universitatsklinikum Hamburg-Eppendorf, Hamburg, Germany

**Keywords:** respiratory biomarkers, human respiratory emissions, environmental monitoring, human respiratory viruses, Human Microbiome Project, machine learning

## Abstract

Humans spend approximately 90% of their lives in built environments, making virus transmission indoors a key determinant of health. Environmental sampling of respiratory viral pathogens is often challenging because of frequent non-detect measurements. Non-detect measurements do not differentiate between samples containing low or no pathogens from samples that simply lack respiratory expulsions altogether. This ambiguity can be resolved by scanning samples for a biomarker of human respiratory emissions. To do so, reliable biomarkers for environmental monitoring need to be identified. Ideal biomarkers are prevalent across individuals, abundant, and unique to the human respiratory tract. Here, we present a new machine learning-based approach to query for suitable biomarker candidates from publicly available metagenomes and apply it to identify viral biomarkers of healthy oral and nasal microbiomes. Twelve viral biomarker candidates were selected from 1,232 curated viral operational taxonomic units. The viral biomarker candidates had as much as 63% prevalence across respiratory metagenomes, and prevalence was further increased to 77%–81% by combining two or three biomarkers. Real-time PCR confirmed that these viral biomarkers were prevalent and abundant in nasal swabs and saliva samples. Notably, top candidate biomarkers remained stable and detectable through multiple lab purification steps, increasing confidence in their viral origins and demonstrating their suitability for environmental monitoring. These findings demonstrate that existing metagenomes can be used to identify effective biomarker candidates for environmental sampling.

**IMPORTANCE:**

Developing non-pharmaceutical interventions to reduce virus transmission indoors relies on robust environmental monitoring methods. Monitoring viral pathogens is challenging because of frequent non-detect measurements that introduce uncertainty. For instance, a non-detect measurement could indicate either the absence of the pathogen or simply the lack of human respiratory activity and, thus, exposure. To aid in distinguishing these scenarios, this study identifies viruses from the human respiratory tract using publicly available sequencing data that can be incorporated into environmental monitoring as biomarkers of human respiratory activity. These viral biomarkers will improve indoor monitoring to help enact interventions to mitigate virus transmission. Furthermore, our approach to identify biomarkers from existing metagenomes can be adapted for future biomarker identification in any system.

## INTRODUCTION

Environmental sampling of indoor spaces is critical to understanding respiratory pathogen transmission and developing effective nonpharmaceutical interventions, especially given that humans spend approximately 90% of their lives indoors (1). Respiratory viruses, such as influenza, coronaviruses, and respiratory syncytial virus, are spread indoors through transmission of droplets (2), aerosols (3), and fomites (4). A challenge of indoor air and surface sampling is the high frequency of non-detect measurements (5–7). Non-detect measurements can arise from multiple scenarios, including (i) no pathogens were shed into the indoor space, (ii) pathogens were shed into the space but were not detected due to issues with the analytical methods (e.g., below detection limit), or (iii) pathogens were shed into the space, but there were issues with the sample collection method (e.g., wrong surface sampled). Differentiating between these non-detect causes is important in transmission studies and in developing effective nonpharmaceutical interventions. To address this challenge, a biological indicator of human respiratory emissions, here termed a respiratory biomarker, is an ideal technical solution. With advances in shotgun sequencing, large repositories of publicly available sequencing data are currently untapped resources for the identification and development of potential biomarkers. A properly selected respiratory biomarker identified from publicly available sequence data could distinguish between pathogen non-detect measurements resulting from limited or no respiratory material in a sample and no pathogen shedding.

Biomarkers play an important role in public health and environmental monitoring by providing reliable indicators of human activity and exposure, thereby enabling accurate detection, assessment, and management of potential contaminants and disease transmission. Biomarkers have been widely implemented for monitoring fecal contamination and for interpreting wastewater epidemiology data. These are typically prokaryotes and viruses with high abundance and prevalence in human populations, such as coliforms (8), enterococci (8), crAssphage (9, 10), and pepper mild mottle virus (11, 12). For example, the United States Environmental Protection Agency bases its fecal pollution indicator policies on the concentration of coliforms and enterococci (13). In wastewater-based epidemiology studies, pepper mild mottle virus and crAssphage concentrations normalize to the proportion of fecal matter in wastewater by accounting for transient changes in population (14). However, these fecal biomarkers were selected without considering if they are found in other areas of humans such as on skin or in saliva. With advances in machine learning and viral bioinformatic tools, viral biomarkers can be identified that are prevalent and unique to particular environments. To date, biomarkers have not been identified for human respiratory emissions.

The most effective respiratory virus biomarker would be bacteriophages commonly found in the human respiratory tract, as their dissemination, persistence, and sampling recovery in air and surfaces would likely mirror those of viral pathogens. Many pathogens, including SARS-CoV-2 and influenza, have distinct tissue tropisms that result in different routes of shedding within the respiratory system (15). Therefore, it is necessary to identify biomarkers that represent different respiratory expulsion fluids, such as nasal mucus and saliva. Furthermore, ideal viral biomarkers will be unique to the human respiratory tract and not found in other human samples, such as in stool or on skin. Lastly, as is the case for crAssphage and pepper mild mottle virus in stool samples, ideal viral respiratory biomarkers will be highly prevalent and abundant in humans, so they are able to be identified consistently in environmental samples.

Here, we developed an approach to identify viral biomarker candidates from existing healthy human metagenomes collected as part of the Human Microbiome Project (16). We created viral operational taxonomic units (vOTUs) from nasal and oral metagenomes and then applied various machine learning approaches that balanced biomarker candidate uniqueness to the human respiratory tract with prevalence and abundance in human oral and nasal metagenomes. Twelve viral biomarker candidates were then quantified in saliva and nasal swabs with real-time PCR and compared to the abundance of three prevalent and abundant saliva bacterial populations previously proposed for use in forensics (17). These viral biomarkers can be applied in future environmental sampling studies, and our approach may be utilized in the future to identify new biomarkers.

## MATERIALS AND METHODS

### Healthy human metagenome data set download and quality control

**Fig 1:**
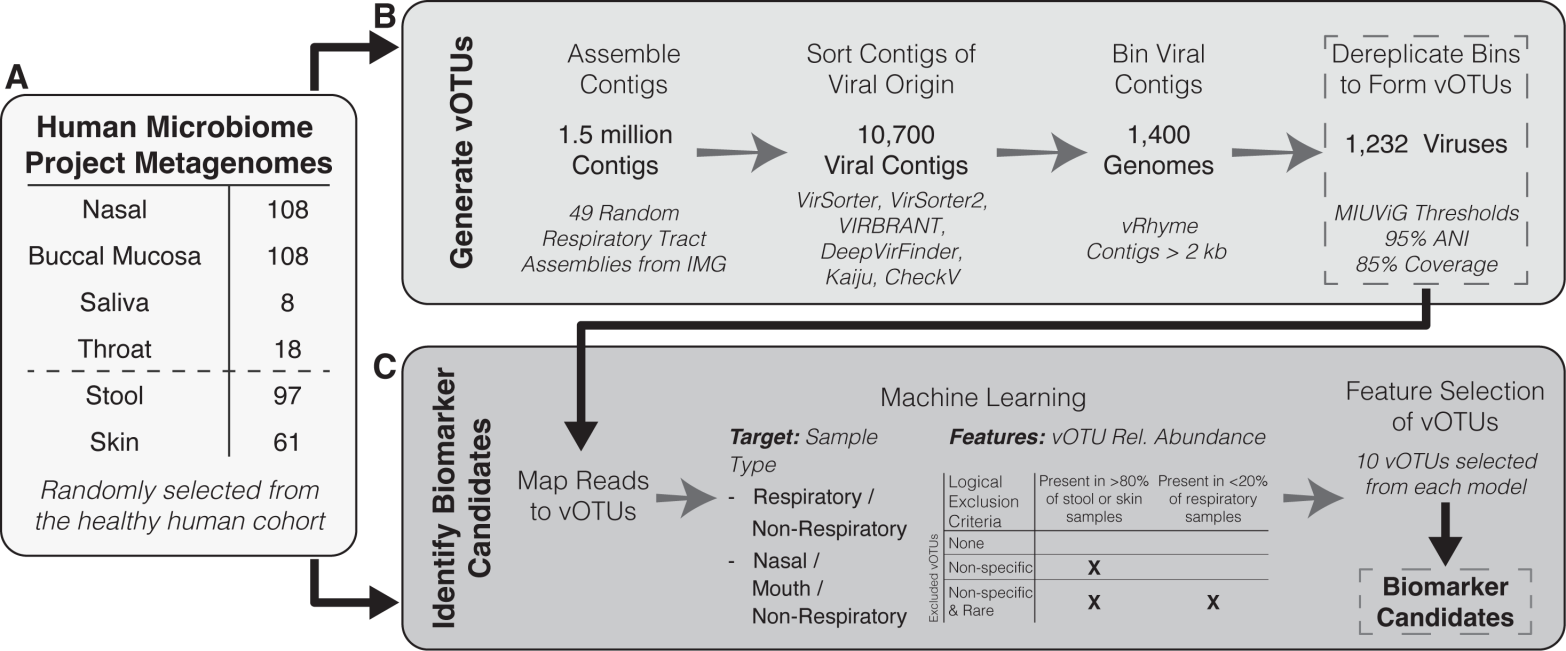
Summary of the process to identify viral biomarker candidates from healthy human metagenomes. (**A**) A random selection of metagenomes from the Human Microbiome Project was used to generate viral operational taxonomic units (vOTUs) and identify biomarker candidates. (**B**) A subset of the metagenomes had their assemblies downloaded from the Joint Genome Institute (JGI) Integrated Microbial Genomes database (IMG) to generate vOTUs. (**C**) Biomarker candidates were identified using machine learning modeling with the relative abundance of vOTUs across the metagenomes from the Human Microbiome Project.

A collection of healthy human metagenomes from the Human Microbiome Project (HMP; https://hmpdacc.org/hmp/) was downloaded from NCBI DbGaP (Project Accession phs000228; Table S1). A subset of available metagenomes was randomly selected using the sample_n function (dplyr, v1.1.4). Samples representing the respiratory tract included 108 anterior nares (i.e., nasal), 108 buccal mucosa, 8 saliva, and 19 throat metagenomes; non-respiratory tract samples included 97 stool and 61 retroauricular crease skin metagenomes (Fig. 1A). Of the metagenomes selected, samples were collected from 164 healthy individuals with 48% (*n* = 78/164) of individuals identifying as female and ages ranging 18–40 years old.

This study is limited in sample type to anterior nares, buccal mucosa, saliva, throat, retroauricular crease, and stool metagenomes available in the healthy human cohort of the HMP. These data were used in order to minimize methodological variability, given HMP’s harmonized sampling and processing protocols, and to remove the influence of dysbiosis and disease, while also ensuring an adequate sample size for machine learning. Ideally, the study would have included nasopharyngeal samples, but this sample site was not included in the HMP study. At the time of the data search for this study (March 2024), there was only one publicly available nasopharyngeal metagenomic data set with 454 pyrosequencing from diseased individuals (18). Furthermore, adequate data from healthy human metatranscriptomes are scarce, which precluded consideration of RNA viruses. Of those that existed during study design, sample sizes were insufficiently small with disparate collection and processing methods used across studies (19–21).

Quality control of reads was performed by trimming Illumina adaptors, and then reads were decontaminated of PhiX174 with BBDuk (BBTools, v37.64) (22). Bases with quality scores less than 10 were trimmed from reads, and then trimmed reads with quality scores less than 10 or lengths less than 100 bp were removed with BBDuk. Human reads were removed using the Human Host Filtration Pipeline (23).

### Generation of viral operational taxonomic units

Assemblies from 49 random respiratory metagenomes (19 nasal, 27 buccal mucosa, 2 throat, and 1 saliva) were downloaded from the Joint Genome Institute (JGI) Integrated Microbial Genomes database (IMG) to curate viral operational taxonomic units (vOTUs) (Fig. 1B; Table S2). To assess the likelihood of each contig being viral, six viral detection methods were run in sequence on contigs longer than 2,000 bp: VirSorter (v1.0.6) (24), VirSorter2 (v2.2.2) (25), VIBRANT (v1.2.1) (26), DeepVirFinder (v1.0) (27), Kaiju (v1.8.0) (28), and CheckV (v1.0.3) (29). Potential viral contigs were identified from these results using previously established rules (30). The viral contigs were binned using vRhyme (v1.1.0) (31) with a minimum contig length of 2,000 bp. The vRhyme bins labeled as “best” bins or circular viral contigs were retained. Viral bins from all of the samples were pooled and dereplicated with dRep (v3.4.3) (32) if bins shared greater than 95% average nucleotide identity and greater than 85% coverage of the shortest contig to form vOTUs (33). Viral sorting, binning, and dereplicating resulted in 1,232 vOTUs (Fig. 1B).

### Read mapping to vOTUs, bacterial saliva biomarkers, and stool biomarkers

Read mapping was performed to assess the prevalence and uniqueness of vOTUs and other targets among the selected healthy human metagenomes (Fig. 1C). We compared the prevalence and abundance of viral biomarker candidates to three commensal oral bacteria and a commonly used fecal biomarker, crAssphage. The three saliva bacteria and crAssphage are known to be highly prevalent and abundant in their respective matrices. The three saliva bacteria were *Streptococcus salivarius*, *Streptococcus sanguinis*, and *Neisseria subflava* (17). To map reads to saliva bacteria, the 3,000 bp region of their genomes including and surrounding previously developed PCR amplicon sequences was downloaded from NCBI (Table S3). The RefSeq crAss-like virus database was downloaded (24 August 2024) to have a reference set of dsDNA viruses commonly used as stool biomarkers (9). Often, a PCR primer assay, CPQ_056 (34), is used in environmental sampling that captures a subset of crAssphage (i.e., NCBI accessions MW063138.1, MW067003.1, MW067002.1, MW067001.1, MW067000.1, MT006214.1, MK415410.1, MK415408.1, MK415404.1, MK415403.1, MK238400.1, NC_024711.1, BK049789.1, MZ130481.1, and MK415399.1). Five Bowtie2 (v2.4.2) indexes were built with default parameters for vOTUs, *N. subflava*, *S. salivarius*, *S. sanguinis*, and crAss-like virus targets. Read mapping with deinterleaved fastq formatted files of QC short reads was performed using Bowtie2 with the default mapping parameters. The number of basepairs mapping to each target was determined from Bowtie2 sam file output using flagstat (samtools, v1.11). The relative abundance of each target was calculated as the ratio of basepairs mapping to each target divided by basepairs in a sample.

### Biomarker candidate selection using machine learning

To identify vOTUs as candidates for human respiratory emission biomarkers, we used machine learning to select vOTUs that best distinguished respiratory samples from stool and skin samples (Fig. 1C). We used supervised classification machine learning with kernelized support vector machines (Python v3.11) to handle our high-dimensional data (35). In total, we created six different models by varying classification structure and logically excluding vOTU features. We utilized two classification structures: (i) respiratory (i.e., nasal, buccal mucosa, saliva, and throat) or non-respiratory (i.e., skin and stool) samples and (ii) nasal, oral (i.e., buccal mucosa, saliva, and throat), and non-respiratory samples. There were three sets of vOTU features used in our machine learning models: (i) the total 1,232 curated vOTUs; (ii) a subset (1,127) of the total vOTUs that excluded “nonspecific vOTUs,” defined as those present in more than 80% of stool or skin samples; and (iii) a subset (703) of the total vOTUs that excluded non-specific vOTUs as well as “rare vOTUs” defined as those that were present in less than 20% of respiratory samples. Models were created to identify respiratory viral biomarkers and were based on the relative abundances of vOTUs in each sample. The data set was divided into training and test sets composed of 279 and 94 samples, respectively. Model accuracy measured the frequency that metagenome origin was correctly predicted. Ten vOTU features that best predicted sample origin classification were identified with forward feature selection. Model accuracy was measured again after feature selection. vOTUs selected as features by more than one model were chosen as viral biomarker candidates of human respiratory emissions. The biomarker candidates were combined to form “cocktails” to assess if having mixes of multiple targets would improve prevalence and abundance in respiratory samples. Every combination of two and three viral biomarker candidates was assessed for prevalence and abundance in respiratory and non-respiratory samples (Fig. S1).

### Bioinformatic confirmation that biomarker candidates were of viral origin

To confirm that all of the selected biomarker candidates were of viral origin, the genomic content was assessed for each contig comprising the vOTUs (Table S4). Two methods were used to assign taxonomy and functional potential. Taxonomy and functional potential were assigned with geNomad (v1.11 with database v1.9) (36) run end-to-end, which performs assignments by combining a neural network-based classifier and protein marker-based classifier. An alignment approach to taxonomic assignment was also run with megaBLASTn to viruses in the NCBI nucleotide database (performed on 19 March 2025). Functional annotation was performed with Pharokka (v1.7.5) (37) using the PHROG reference database (performed on 3 August 2025) and PHANOTATE to predict genes along contig sequences. To establish confidence in viral provenance, we leveraged the knowledge that viruses have compact genomes with high coding density (38). The coding density of vOTUs was calculated from the PHANOTATE results as the total sequence length within open reading frames divided by the total sequence length. If taxonomy, functional potential annotation, and coding density resulted in uncertainty in whether the vOTU was a bonafide virus, further alignments were performed (on 1 April 2025) with BLASTn to the NCBI core nucleotide database, viruses in the NCBI Whole Genome Shotgun (WGS) contigs, and bacteria in the NCBI RefSeq Genome databases. Once the biomarker candidates were confirmed as viruses, the vOTUs were renamed based on where their prevalence was highest in respiratory environments (O = oral; N = nasal) and ranked based on prevalence and abundance in the metagenomic and real-time PCR (see details below) data sets.

### Laboratory confirmation that biomarker candidates were of viral origin

Viral particles were purified from pooled saliva and pooled nasal mucus to provide physical evidence that the selected biomarkers were of viral origin. Freshly collected unstimulated 1-mL saliva and nasal swabs were collected from three individuals as approved by the Institutional Review Board (IRB-HSBS) at the University of Michigan (HUM00241431). Saliva samples were collected with SDNA-1000 saliva collection kits (Spectrum Solutions) without the stabilization buffer added, and nasal swabs were collected with Quickvue Influenza Nasal Swab Tubes (Quidel). The three saliva samples were pooled, diluted with 7 mL of filter-sterilized PBS with 0.5% bovine serum albumin, and then vortexed until homogenous. The nasal swabs were combined with 2 mL of PBS containing 0.5% bovine serum albumin and vortexed for 1 min. A 400-µL aliquot of each saliva and nasal mucus sample was stored at 4°C until DNA extraction.

The remaining 9.6 mL of saliva and 1.6 mL of nasal mucus samples were filtered through a 0.22-µm PES syringe filter (Millipore Sigma, Cat. No. SLGPR33RS) in order to remove large, non-viral particles such as bacteria and cells. A 400-µL aliquot of each filtered saliva and nasal mucus sample was removed and stored at 4°C until DNA extraction. Chloroform was then added to the remaining sample volumes to lyse cells while not compromising most viral protein capsids with the exception of some enveloped viruses (39) and filamentous viruses (40). Specifically, 500 µL and 100 µL of chloroform were added to the filtered saliva and nasal mucus, respectively, and then, the samples were vortexed for 2 min. After setting for 15 min, the chloroform was removed from the sample by first pipetting the chloroform layer and then by gently aerating the sample. Aeration reduced the volume of the samples; therefore, the nasal mucus samples were re-diluted to a volume of 800 µL with PBS containing 0.5% bovine serum albumin. A 400-µL aliquot of each chloroform-treated saliva and nasal mucus sample was collected and stored at 4°C until extracting DNA.

Finally, non-encapsulated DNA was removed from the remaining sample volumes by adding 100 U/mL DNase I (Sigma Aldrich, Cat. No. 10104159001) that was suspended in a reaction buffer containing 750 µM Tris-HCl, 200 µM MgCl_2_, and 40 µM CaCl_2_. The DNase was allowed to react with the saliva and nasal mucus on the benchtop for 1 h and then heat inactivated at 75°C for 5 min. Final 400-µL aliquots of DNase-treated saliva and nasal mucus were collected.

### Saliva and nasal swab collection

Saliva and nasal swab samples were collected from 10 individuals to study the prevalence and abundance of the candidate biomarkers with real-time PCR. Unstimulated saliva and nasal swabs were collected in the evening from 10 individuals with no self-identified chronic respiratory diseases over the age of 18. Saliva samples were collected with SDNA-1000 saliva collection kits (Spectrum Solutions) and nasal swabs were collected with Quickvue Influenza Nasal Swab Tubes (Quidel) with 1 mL of PBS with 0.5% bovine serum albumin added. The study and all associated documents and protocols were approved by the IRB-HSBS at the University of Michigan (HUM00241431). Samples were stored at −80°C for a maximum of 36 days until DNA extraction.

### DNA extraction

Samples were thawed on ice immediately prior to DNA extraction. DNA was extracted in duplicate from 200 µL sample aliquots with a Kingfisher Flex instrument equipped with a 96-well attachment. The Applied Biosystems MagMAX Viral/Pathogen II Nucleic Acid Isolation Kit (Fisher Scientific Cat. No. A48383) was utilized with two wash cycles and 50 µL elutions. DNA extracts were stored at −20°C for a maximum of 1 week until real-time PCR was performed.

### Biomarker candidate PCR primer design and real-time PCR conditions

The presence and relative abundance of viral biomarker candidates and bacterial saliva biomarkers were assessed in saliva and nasal swab DNA extracts using real-time PCR. Primers for the 12 vOTUs selected as viral biomarker candidates were designed using the IDT PrimerQuest tool (Integrated DNA Technologies). The best primer assay for each vOTU was selected to maximize specificity. Specificity of each primer assay was assessed with NCBI Primer BLAST to the nr database where no or few hits indicated high specificity. Primer sequences for the selected viral biomarker candidates and previously identified saliva bacterial biomarkers (17) primers are provided in Table S5. We conducted real-time PCR on both the pooled samples that had been enriched for viruses (i.e., post-filtration, post-chloroform, and post-DNase treatment) to confirm that the biomarkers were of viral origin and the saliva and nasal swabs from 10 individuals to assess presence and abundance of the candidate biomarkers. Reactions were performed on each of the duplicate DNA extracts with the QuantStudio 3 thermocycler (Thermo Fisher Scientific, Inc.). For each plate, two ddH_2_O negative controls for each primer set were analyzed with the samples. The 20 µL reactions were prepared with 10 µL of Luna Universal qPCR mastermix (New England Biolabs, Cat. No. M3003), 0.5 µM of forward and reverse primers, and 5 µL of 1:10 diluted template. The reactions consisted of initial denaturation at 95°C for 60 s followed by 45 cycles of denaturation for 15 s at 95°C, then annealing and extension for 30 s at 60°C. The mean real-time PCR threshold cycle (Ct) of duplicate reactions for each biomarker in the DNA extracts was used in the analysis. The biomarker was not detected in a sample if the mean Ct value was less than or equal to the mean NTC from the specific plate run. These real-time PCR measurements for all viruses and bacteria are a proxy for quantification due to the unavailability of standard curves for each target.

### Statistical analysis

All statistical analysis and figure creation was performed with R (v4.4.0) using ggplot2 (v3.5.1) and plotly (v4.10.4). Normality was tested with the Shapiro-Wilkes test. Wilcoxon’s test with Benjamini–Hochberg’s correction was performed on non-normal data sets.

## RESULTS AND DISCUSSION

### Oral metagenomes generated more vOTUs than nasal metagenomes

To identify viruses pervasive in human respiratory emissions, we curated 1,232 vOTUs from 49 healthy human oral and nasal metagenomes (Fig. 1B). The oral samples (i.e., buccal mucosa, saliva, and throat) had significantly more assembled viruses than nasal samples, with oral and nasal assemblies having an average of 45 viral genomes and 2 viral genomes, respectively (*P*-value = 1.4 × 10^−7^; Table S2). A previous analysis of viruses in samples from the Human Microbiome Project (HMP) identified significantly more vOTUs in oral than nasal samples (41). This observation may be due to fewer viruses comprising the nasal microbiome or issues with the sequencing data quality. In our study, quality-controlled sequencing depth was significantly greater in oral metagenomes than nasal metagenomes (*P*-value = 2.6 × 10^−5^). The oral assemblies also contained significantly more contigs longer than 1,000 bp than nasal assemblies (*P*-value = 4.9 × 10^−7^). Despite the nasal assemblies resulting in fewer contigs, the median contig lengths were not significantly different between oral and nasal assemblies (*P*-value = 0.26), indicating similar assembly quality between oral and nasal metagenomes. Previous comparisons of oral and nasal microbiomes are inconsistent as to whether the oral microbiome is more diverse than the nasal microbiome (42–45). Diverse viral communities have been reported in the oral cavity (41, 46–49). However, the few prior studies on nasal cavity viruses resulted in a small number of vOTUs, suggesting low viral richness (41, 50).

### Twelve vOTUs were selected as viral respiratory biomarker candidates

We next identified candidate viral biomarkers of human respiratory emissions from the vOTUs. Ideal viral respiratory biomarkers would be highly abundant and prevalent in respiratory samples and not found in other human environments, such as in stool or on skin. Ideal candidates were identified based on three criteria: (i) machine-learning identification of vOTUs associated with oral, nasal, skin, or stool samples, (ii) evaluation of vOTU abundance and prevalence across target (i.e., oral and nasal) and non-target (i.e., stool and skin) body sites, including consistency of selection across multiple machine learning models, and (iii) agreement among machine learning models on the best respiratory vOTU biomarker candidates.

**Fig 2:**
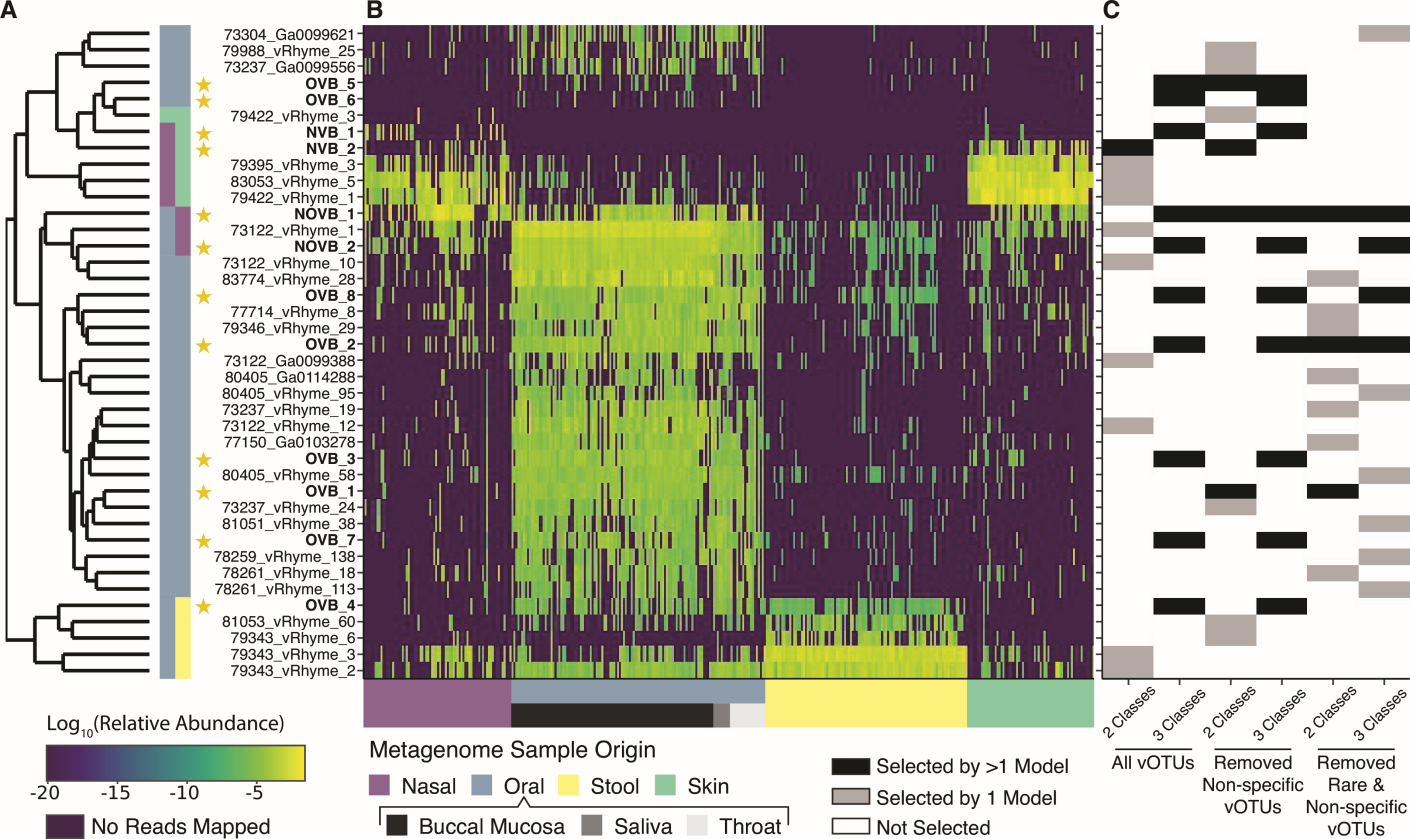
Relative abundance of vOTUs selected as key features in machine learning models across the HMP metagenomes. (**A**) vOTUs are organized by hierarchical clustering with Euclidean distances based on relative abundances across all HMP metagenomes. The vertical color bar denotes clusters of vOTUs indicative of particular sample origins where nasal, oral, stool, and skin are represented by purple, blue, yellow, and green, respectively. The vOTUs identified by more than one model were selected as viral biomarker candidates, indicated with a star and bolded name. (**B**) Logarithmic relative abundance of vOTUs in each sample, where dark purple indicates a vOTU was not present in a Human Microbiome Project metagenome. The metagenome sample origin is indicated by the *x*-axis color bar. (**C**) Heatmap indicating which vOTUs were selected during feature selection in each of the kernelized support vector machine learning models. The models had two classification options to determine sample origins: two classes (respiratory or non-respiratory) and three classes (nasal, oral, or non-respiratory). There were three sets of features used: all 1,232 vOTUs, 105 non-specific vOTUs removed, and 529 rare or non-specific vOTUs removed (Fig. 1C).

Six machine learning models were applied to predict the origin of the 400 metagenomes based on the relative abundances of vOTUs across respiratory (i.e., oral and nasal) and non-respiratory (i.e., stool and skin) metagenomes. In order to select biomarker candidates from the vOTUs, we performed modeling using three sets of increasingly filtered vOTU features: all 1,232 vOTUs, a subset of the vOTUs with the non-specific vOTUs removed (i.e., vOTUs present in more than 80% of stool or skin samples), and a subset of the vOTUs with the non-specific and rare vOTUs (i.e., vOTUs present in less than 20% of oral and nasal samples) removed. Across the six machine learning models, the accuracies ranged from 0.49 to 0.85 (Table S6); given these values were less than 0.95−1, this indicated that our models were not overfitted to the training data. We next applied feature selection to identify the 10 vOTUs that best predicted sample origin from each model. The models were rerun using only the selected 10 vOTUs, and this resulted in improved accuracy ranging from 0.70 to 0.93 (Table S6). Across the 6 models, 40 different vOTUs were selected as key features by at least one model (Fig. 2A).

We next evaluated the abundance and prevalence of these 40 key feature vOTUs in respiratory (i.e., nasal and oral cavities) and non-respiratory (i.e., stool and skin) samples. Of the 40 candidate vOTUs, 24 were highly abundant and prevalent in the oral environment exclusively (Fig. 2A; Table S7). Previous research on bacterial communities has similarly shown that the oral microbiome is distinct from the other human microbiomes (42, 51) though published studies on the viral component of these human microbiomes are limited.

Of the remaining 16 candidates, 3 candidate vOTUs that were prevalent in both nasal and oral samples (i.e., NOVB_1, 73122_vRhyme_1, NOVB_2; Fig. 2A and B). Another three of the remaining vOTUs were highly unique to nasal (i.e., 79422_vRhyme_3) or oral samples (i.e., OVB_5 and OVB_6) but were not considered prevalent in any human environment (<15% prevalence in all sample types; Fig. 2A and B). The remaining 10 candidate vOTUs were poor candidates for biomarkers of respiratory emissions given their high prevalence on skin or in stool: 5 vOTUs were prevalent in nasal and skin samples with 5 other candidate vOTUs prevalent and abundant in oral and stool samples (Fig. 2A and B). Although these skin and stool vOTUs aided models in determining sample origin, they are not ideal respiratory biomarker candidates.

The identification of these vOTUs prevalent in stool reinforced the decision to incorporate logical exclusion criteria in four of the models to exclude vOTUs that were highly prevalent in the non-target stool or skin samples (Fig. 2C). The overlap of vOTUs in skin and nasal metagenomes aligns with previous findings of similar microbiota in skin and nasal samples (42). Likewise, our observations of some correspondence between stool and oral vOTUs are not surprising, given prior observations of overlap between the oral and distal colon microbial communities (52), which have given rise to hypotheses that the oral microbiome may seed the gastrointestinal tract (53).

We next looked for agreement between the six machine learning models in order to narrow which vOTUs are possible biomarkers. Twelve of the 30 vOTUs remaining as ideal candidates were selected as key features by more than 1 model (Fig. 2C; Table S4). The two most often selected vOTUs, NOVB_1 and OVB_2, were identified by five and four models, respectively. NOVB_1 and OVB_2 were commonly found in oral samples with NOVB_1 also found in nasal samples. The remaining vOTUs were selected by two (*n* = 7) or three (*n* = 3) models with the greatest prevalence in oral samples (*n* = 4), nasal and skin samples (*n* = 2), nasal and oral samples (*n* = 1), or oral and stool samples (*n* = 1). Two vOTUs, OVB_5 and OVB_6, were highly specific to oral samples although they were rarely present in the metagenomes. Given that these 12 vOTUs were identified by more than 1 model, all were further evaluated as viral biomarker candidates in this study.

### Biomarker candidate genomes demonstrate evidence of viral origins

We next evaluated whether the 12 biomarker candidates identified were of viral, rather than of bacterial or human origins. All contigs that comprised the 12 candidate vOTUs were confidently identified as being of viral origin based on a previously developed and published viral contig sorting algorithm that utilizes 6 viral detection methods to score potential viral contigs (30). To further increase our confidence in this viral assignment, we sought additional evidence, including (i) viral taxonomy assignment with multiple methods, (ii) identification of known viral proteins on the contigs, or (iii) a high coding density characteristic of viral, rather than cellular, genomes. Based on our taxonomy assignment approach, all 12 biomarker candidates were assigned as viruses by at least 1 method, except for NOVB_1 and OVB_1 (Table S4). Viral proteins, or those known to be associated with viral functions, were identified on the contigs of the biomarker candidates, with the exception of NOVB_1 (Table S4). The coding density of the vOTUs was high (90%–98%; Table S4) for 11 biomarker candidates, exceeding that of the human genome (1%–2%) (54), and consistent with viral and bacterial origins (38, 55). NOVB_1 was the exception, with a lower coding density of 71% (Table S4). Combined, these results suggested that all candidate biomarkers except NOVB_1 fit expectations of viral origins based on taxonomic assignment, gene annotation, and coding density.

To test if the 12 viral biomarker candidates were associated with virus-like particles in human oral and nasal samples, we conducted various virus enrichment steps on freshly collected saliva and nasal mucus samples from three individuals. Specifically, we subjected the samples to three sequential viral purification methods, including size exclusion filtration, chloroform lysis, and DNase degradation of non-encapsulated DNA. Prior to any enrichment, all but NVB_1 and OVB_8 were present in the pooled saliva or pooled nasal samples (Table S8). Size exclusion filtration tested if the viral biomarker candidates could pass through 0.22-µm pores, which is a commonly applied size threshold for viral particles (56, 57). Of the remaining 10 biomarkers, all but OVB_7 were present in the filtrate (Table S8), indicating that most of the biomarker candidates fall within the conventional viral size range. Chloroform treatment disrupts lipid-containing cell membranes, while leaving viral protein capsids intact—though a few virus types, such as enveloped viruses (39) and filamentous phages (40), can be chloroform-sensitive. After chloroform treatment, 8 of the remaining 9 biomarker candidates were measurable in at least 1 of the samples (Table S8). OVB_5 was not detected following chloroform treatment. Following DNase treatment, only three of the biomarker candidates remained detectable, namely, NOVB_1, OVB_1, and OVB_4 (Table S8). The lack of the detection of certain viral biomarker candidates after the chloroform and DNase treatment could be due to these candidates having capsids susceptible to chloroform, having most of their DNA free or in compromised capsids prior to DNase treatment, or low initial concentrations that fell below detection thresholds after the purification steps or dilution. Notably, NOVB_1 and OVB_1 were measurable after each purification step in both saliva and nasal mucus.

We further evaluated the contigs comprising the NOVB_1 vOTU, the only biomarker without any viral genes annotated, to determine whether the putative vOTU was misassigned as viral, or if instead it represented novel, uncharacterized viral diversity, referred to as “viral dark matter” (58, 59). Methods for identifying viral dark matter rely on deep learning methods, such as DeepVirFinder (60), and alignments to viral contigs from shotgun sequencing (61). DeepVirFinder indicated that both NOVB_1 contigs were of viral origin (*P*-values = 0.032 and 0.033). When NOVB_1 was aligned to the core nucleotide database (BLASTn), it aligned best to the *Homo sapiens* chromosome 5 (NCBI accession OZ171101.1; bit score = 10,761). However, we also searched for homology in the whole genome sequencing database (NCBI WGS) and identified that the NOVB_1 contigs aligned best to two assembled phage contigs (bit scores = 73.4 and 66.2). The NOVB_1 contigs were assembled from an anterior nares metagenome with human contamination accounting for 4% of reads. It is possible that NOVB_1 is related to a non-retroviral endogenous virus; however, gene annotation did not identify any genes for chromosomal integration (62). Additionally, NOVB_1 was not universally present across the metagenomes as would be expected for human genome fragments but primarily present in oral and nasal metagenomes (Fig. 2B). Based on this evidence, combined with its high coding density (71%) relative to that expected of the human genome (1%–2%) (54) and that it was measurable after three viral purification steps, we concluded that NOVB_1 is a novel virus sequence that represents undescribed and uncharacterized sequence space of “viral dark matter.”

Machine learning feature selection utilized the prevalence and relative abundances of the vOTUs to select key viruses indicative of respiratory samples; however, these analyses do not evaluate the performance of the selected vOTUs as biomarkers. The next 2 sections assess the performance of the 12 candidate biomarkers by examining their prevalence and relative abundance in the respiratory microbiomes compared to the other sample types. We also compare the performance to previously identified bacterial respiratory biomarkers, *N. subflava*, *S. salivarius*, and *S. sanguinis* (17), and a fecal viral biomarker, crAssphage (9).

### The prevalence of viral biomarker candidates across respiratory and non-respiratory samples

**Fig 3:**
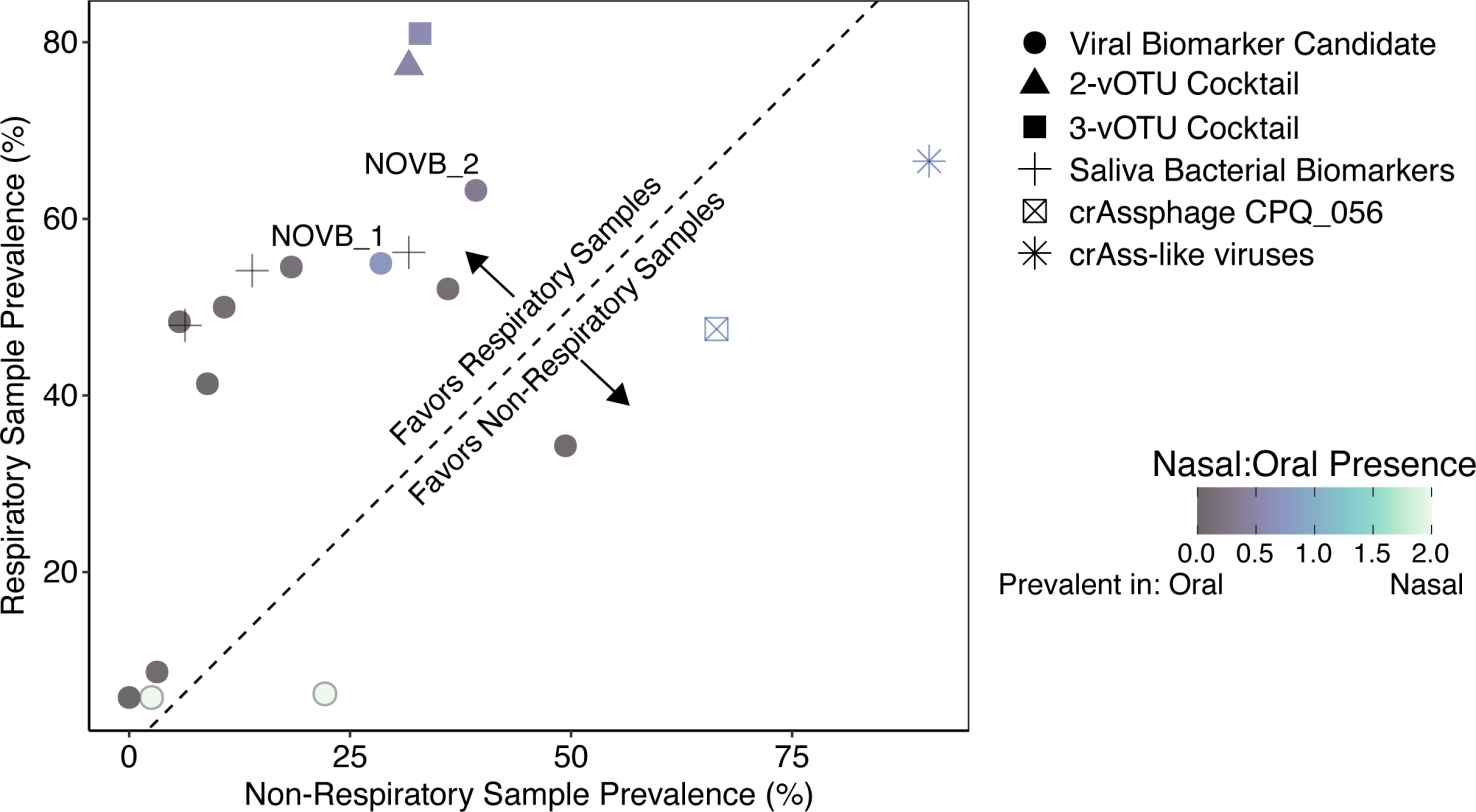
The prevalence of viral biomarker candidates, best viral biomarker candidate cocktails, three saliva bacteria from Jung et al. (17), and crAssphage are compared across the different sample origins. The best viral biomarker candidate cocktails included a two-vOTU cocktail (NOVB_1 and OVB_1) and a three-vOTU cocktail (NOVB_1, OVB_1, and NVB_1). The points indicate the percent of samples with a target present in non-respiratory compared to respiratory samples. The dashed 1:1 line divides which targets are more prevalent in respiratory or non-respiratory samples. Points are shaped by viral biomarker candidates, two-vOTU cocktail, three-vOTU cocktail, saliva bacterial biomarkers, crAssphage CPQ_056 amplicon, and all crAss-like viruses as circles, triangles, squares, plus sign, crossed box, and asterisk, respectively. Points are colored based on the ratio of the prevalence in nasal compared to oral samples.

The prevalence of the 12 biomarkers across the respiratory samples ranged from 6% to 63%. Of the 12, 10 were more prevalent in respiratory samples (nasal and oral) than non-respiratory samples (stool and skin) (Fig. 3; Table S9). Specifically, NVB_2 and OVB_4 were prevalent in skin and stool, respectively. Five of the viral biomarker candidates were present in the majority of respiratory samples (range = 50%–63%) and were less commonly found in non-respiratory samples (range = 11%–39%). Of these five biomarker candidates with the highest prevalence in respiratory samples, three were specific to oral samples and two were found in both oral and nasal samples (Table S9). Both of the nasal-specific viral biomarkers, NVB_1 and NVB_2, were relatively rare (less than 10% prevalence) across respiratory samples (Fig. 3). Combined, these results suggest that several of the candidate biomarkers may be widespread in human respiratory microbiomes and expected to perform well as respiratory biomarkers.

The prevalence of the five viral biomarkers specific to oral samples was similar to that of the three saliva bacteria that were previously identified as forensics saliva biomarkers (17) (Fig. 3). The saliva bacteria were present in 48%–56% of respiratory samples and 6%–32% of non-respiratory samples. When combined, the saliva bacteria biomarkers were more prevalent in oral (*n* = 133/134) than nasal (*n* = 23/108) samples. CrAssphage was highly prevalent in respiratory (67%) and non-respiratory (91%) samples, with the prevalence of all crAssphage exceeding the most prevalent viral biomarker candidate, NOVB_2, in respiratory metagenomes (Fig. 3). crAssphage CPQ_056 primers (34) and all crAss-like viruses were not specific to stool with high prevalence across all human microbiome samples. This was unexpected because crAssphage is commonly used as a human fecal biomarker. Previously, Tisza et al. (41) reported that most viruses are found at only one body site, with only ~5% viruses found in at least two sites, including some crAss-like viruses (41). This report of multi-site distribution is consistent with our own findings, but was surprising nonetheless, given crAssphage is an established human fecal indicator. However, this observation may have been overlooked given that previous studies investigating its habitat specificity focused on its potential as a fecal indicator in water quality applications, thus emphasized its presence in the human gut relative to other animals or environments, rather than other human body sites (9, 10, 63–67).

Given observed variations in the human microbiome across individuals (52, 68) and our finding that no single vOTU was present in every metagenome, it is unlikely that every person in a shared space over a given time frame will have a universally shared vOTU in their respiratory fluids. Given this expected variability among individuals, we assessed whether grouping multiple viral biomarker candidates into cocktail mixtures could capture a wider population with higher prevalences. Specifically, we conducted an *in silico* assessment of two- and three-vOTU cocktails for the 12 candidate respiratory biomarkers (Fig. S1).

Based on their prevalence across all of the metagenomes, we identified a two-vOTU cocktail and a three-vOTU cocktail that showed high prevalence in respiratory samples (77% and 81%, respectively) and low prevalence in non-respiratory samples (32% and 33%, respectively; Fig. 3). Both cocktails included NOVB_1, a prevalent biomarker in oral and nasal metagenomes, and OVB_1, a highly prevalent biomarker in oral metagenomes (Fig. 2B). The three-vOTU cocktail also included NVB_1, which was unique to the nasal cavity (Fig. 2B). NOVB_1 alone was found in 63% of oral metagenomes, whereas the two-vOTU cocktail was found in 99% of oral metagenomes. In the nasal metagenomes, the three-vOTU cocktail was present in 58% of samples, compared to 46% for NOVB_1 alone. Both cocktails had minimal impact on prevalence in non-respiratory samples, with the two-vOTU and three-vOTU cocktail being present in 3% and 4% more of non-respiratory metagenomes, respectively. In practice, these cocktails could be measured by performing separate quantitative PCR assays for each biomarker or as a single quantitative assay with a mixture of the primers to reduce supplies and costs. To facilitate the latter approach, we designed primer sets for the viral biomarker candidates to have uniform reaction conditions and similar amplicon lengths (Table S5). Given these findings, the use of a viral respiratory biomarker cocktail can help account for the inter-individual variability in respiratory virus community composition and thereby reduce false negatives that would be expected if only one vOTU was used as a marker of human respiratory emissions.

### Viral biomarker candidates had similar abundance to accepted standards

**Fig 4:**
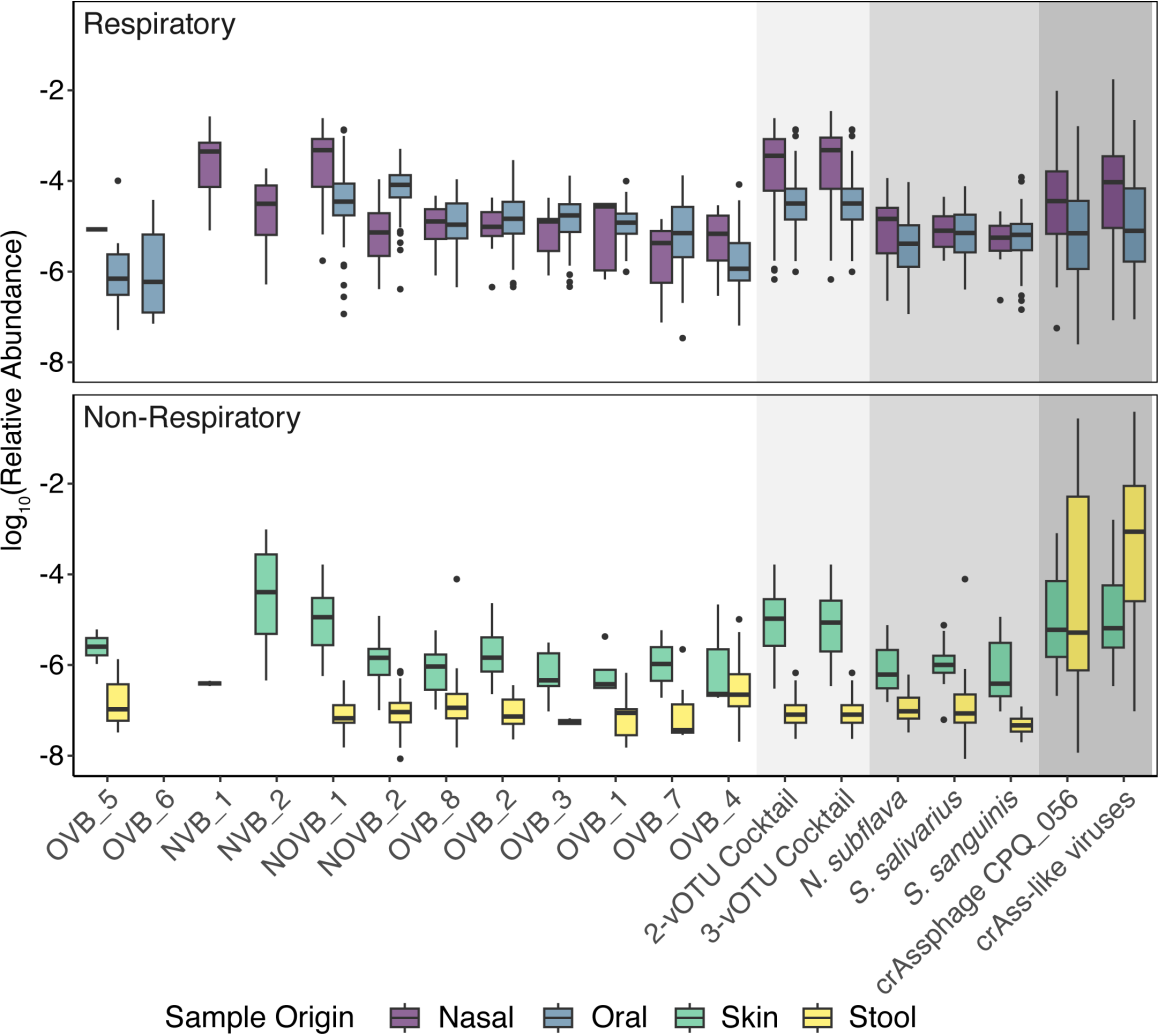
The abundance of viral biomarker candidates, best viral biomarker candidate cocktails, three saliva bacteria from Jung et al. (17), and crAssphage are compared across the different HMP metagenome sample origins. The best viral biomarker candidate cocktails included a two-vOTU cocktail (NOVB_1 and OVB_1) and three-vOTU cocktail (NOVB_1, OVB_1, and NVB_1). Boxplot of logarithmic relative abundances for targets across the different sample origins where nasal, oral (buccal mucosa, saliva, and throat), skin, and stool are represented in purple, blue, green, and yellow, respectively. Any instances where a target was not present in a sample were not included.

Ideally, respiratory biomarkers would be both prevalent and abundant in respiratory samples. Therefore, we assessed the relative abundances of the viral biomarkers and the cocktails in the metagenomes. The mean relative abundances of viral biomarkers ranged 0.000431%–0.0106% in oral metagenomes and 0.000624%–0.0585% in nasal metagenomes (Fig. 4). All 12 of the viral biomarker candidates had a greater mean relative abundance in respiratory samples than non-respiratory samples with 10 of those differences being statistically significant (*P*-values < 7 × 10^−4^; Fig. 4; Fig. S2A). The two that were not statistically greater in respiratory samples than other samples (OVB_5 and NVB_2), exhibited low prevalence in the respiratory samples (Fig. 3; Table S9). NOVB_1 had the greatest mean relative abundance in oral (0.0106%; *n* = 84/108) and nasal (0.0585%; *n* = 49/108) samples (Fig. 4; Fig. S2A). Although the cocktails increased prevalence in respiratory samples across individuals, the combined abundances of vOTUs in the cocktails were not significantly greater than NOVB_1 alone in any sample type (*P*-values = 0.53–0.94; Fig. 4). In summary, several viral biomarker candidates were highly abundant in either nasal or oral cavities, with NOVB_1 abundant in both environments, indicating its potential as a marker for respiratory monitoring.

When present, in the respiratory samples, the mean relative abundances of the saliva bacterial biomarkers ranged from 0.00104% to 0.00139% in oral metagenomes and 0.000744% to 0.00242% in nasal metagenomes. These were within the same range of the viral biomarker candidates’ relative abundances in the respiratory samples (0.000431%–0.0585%). The relative abundances of each of the three bacteria were similar in oral and nasal metagenomes; however, as mentioned above, the saliva bacteria are less frequently present in nasal metagenomes. Two of the viral biomarkers were significantly more abundant than *N. subflava*, the most abundant saliva bacteria biomarker in the nasal metagenomes (*P*-values = 2.0 × 10^−6^ and 8.7 × 10^−4^ for NOVB_1 and NVB_1, respectively; Fig. S2B), and the other nine viral biomarkers present in nasal metagenomes were not statistically different in abundance than *N. subflava*. Whereas, in oral metagenomes, two of the viral biomarkers were significantly more abundant than *S. salivarius*, the most abundant bacterial biomarkers in the oral metagenomes (*P*-values = 1.5 × 10^−12^ and 6.3 × 10^−30^ for NOVB_1 and NOVB_2, respectively; Fig. S2B).

CrAss-like viruses were abundant in the respiratory samples (0.0223%). In oral metagenomes specifically, the mean relative abundance was 0.00992%, and in nasal metagenomes, the mean relative abundance was 0.0742% (Fig. 4; Fig. S2B and C). Compared to the viral biomarker candidates, the mean relative abundance of crAss-like viruses was more abundant than all of the viral biomarkers but one virus in the oral metagenomes. NOVB_1 was significantly more abundant (*P*-values = 2.1 × 10^−4^) than crAss-like viruses in the oral metagenomes. The high abundance of crAssphage compared to the individual vOTUs in this study may be partially due to the multiple populations comprising crAssphage (e.g., 268 crAssphage NCBI accessions).

### Viral biomarkers were found in saliva and nasal swab samples with real-time PCR

**Fig 5:**
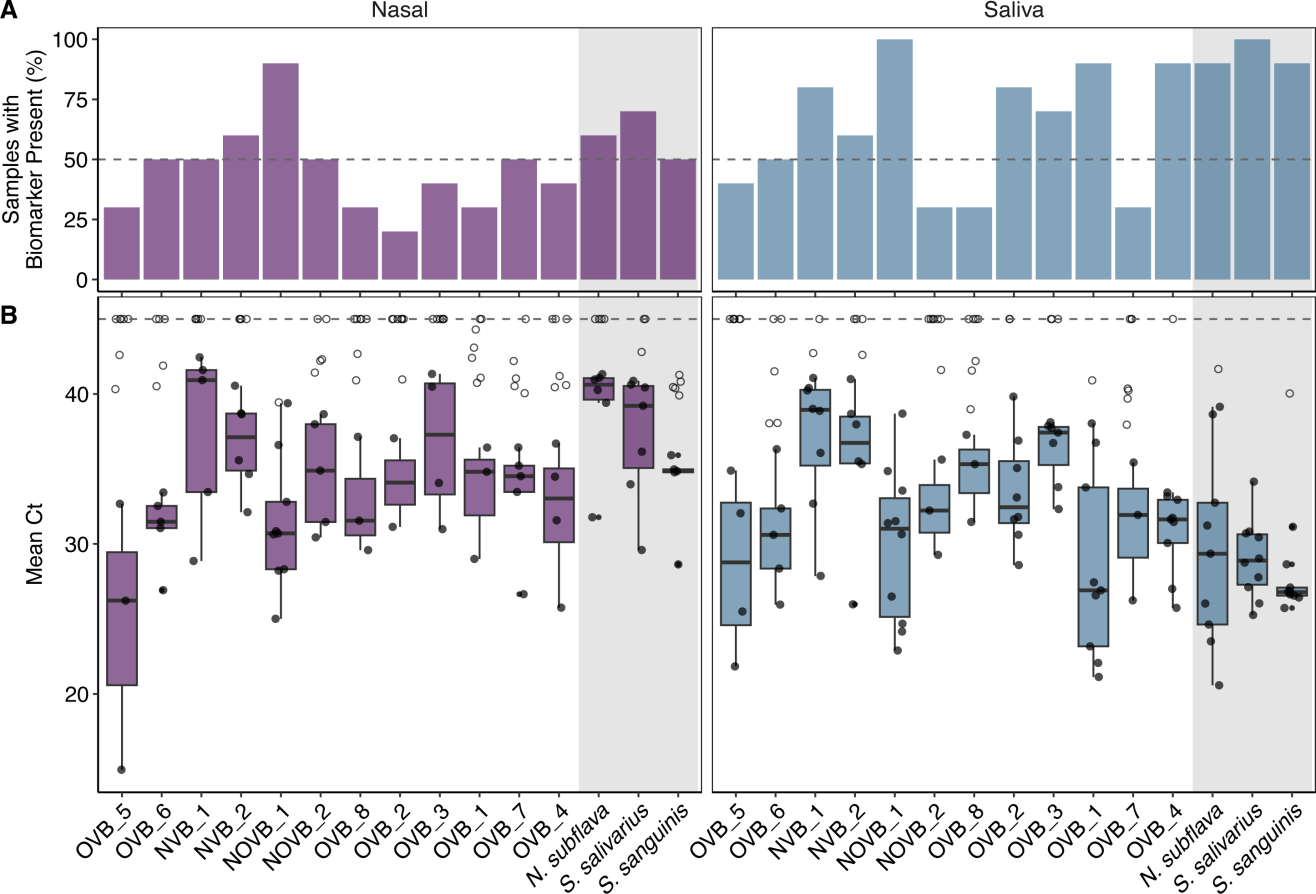
Presence and abundance of human respiratory emission biomarker candidates in nasal swab (*n* = 10) and saliva (*n* = 10) samples. (**A**) Percent of samples present for each biomarker candidate where present is defined as a greater than onefold increase from the negative controls. The dashed line denotes when at least half of the samples had a biomarker present. (**B**) Boxplot of the mean Ct measurements among the samples with the biomarker present. The solid and open circles denote detected and non-detected measurements, respectively, with the dashed line indicating the maximum Ct value of 45. The gray regions contain results for the bacterial saliva biomarkers from Jung et al. (17).

We next tested the ability of real-time PCR to detect the 12 candidate biomarkers in freshly collected saliva samples (*n* = 10) and nasal swabs (*n* = 10; Fig. 5). For the 12 candidates, the prevalence ranged from 30% to 100% in the saliva samples and ranged from 10% to 100% in the nasal swab samples. Five of the viral biomarkers were present in at least 80% of saliva samples, with NOVB_1 found in all of the saliva samples (Fig. 5A). Similarly, NOVB_1 was detected in each of the nasal swab samples; the next most prevalent candidate biomarkers were NVB_1, NVB_2, OVB_5, and OVB_6 found in 50% of the samples. By comparison, the bacterial biomarker prevalence in the saliva samples was 100% for *S. salivarius*, and 90% for *N. subflava* and *S. sanguinis* (Fig. 5A). In the nasal swabs, the bacterial biomarker prevalence was 40% for *S. salivarius* and 30% for *N. subflava* and *S. sanguinis* (Fig. 5A).

Of the biomarkers present in at least 80% of saliva samples, OVB_1 and NOVB_1 were the most abundant with mean Ct values of 27.2 and 29.9, respectively. The three saliva bacteria biomarkers also had mean Ct values less than 30 cycles in saliva. In nasal swabs, no viral or bacterial biomarker had a mean Ct value less than 30 cycles. OVB_5, NOVB_1, and OVB_6 had the lowest mean Ct values of 31.4, 32.2, and 32.9 cycles, respectively, in nasal swabs. The high abundance of NOVB_1 in saliva and nasal swabs aligned with the *in silico* high relative abundance observations in the metagenomes. Overall, the targets had lower Ct values in saliva samples, which may be attributed to higher overall biomass collected given the methodology. In this study, we did not use probes for the saliva bacteria targets in our real-time PCR assays. This reduces the specificity of the assays based on NCBI primer BLAST results and may have inflated the real-time PCR abundances.

### Conclusion

By assessing the prevalence and abundance of viruses in existing human metagenomes, we identified several viral biomarker candidates with high abundances and prevalences in newly collected nasal swabs and saliva. Based on the combined results, NOVB_1 is the most promising virus for general respiratory biomarkers (i.e., both oral and nasal). In nasal samples, NOVB_1 and NVB_1 were highly prevalent based on both the metagenomes and the nasal swab samples analyzed by real-time PCR. Neither NOVB_1 nor NVB_1 was unique to the nasal environment based on the real-time PCR results despite metagenomic results indicating NVB_1 was unique to nasal samples. Whereas in oral samples, NOVB_1, OVB_1, and OVB_4 were prevalent among the metagenomes and the saliva samples analyzed by real-time PCR. NOVB_1, NVB_1, and OVB_1 were not prevalent or abundant across the skin and stool metagenomes; however, OVB_4 was commonly found in stool metagenomes. Of these, NOVB_1 is the most promising viral respiratory biomarker candidate for environmental sampling due to its prevalence, specificity to the respiratory tract, and high abundance across the metagenomes and real-time PCR results.

Since pathogens are disseminated in air through droplets and aerosols (2, 3) generated from respiratory activities, pathogen-containing particles may originate from one of multiple locations in the respiratory tract, such as the oral cavity or nasopharynx. While influenza is shed from both saliva and nasal mucus, the route of expulsion can impact the transmissibility of the virus. The persistence of the virus may differ depending on the expulsion route and specifically the chemical composition of the matrix (69), and its transport in the environment is likely influenced by differences in particle sizes generated from different expulsion routes (70, 71). Therefore, identifying viral biomarkers unique to different particle origin sites would provide additional information on the persistence and transmission of pathogens in the environment.

Future work will assess the prevalence and abundance of these viral biomarker candidates on masks, surfaces, and air filters. Additional investigations into combining multiple viral biomarkers to capture a wider population and increase the total abundance will also be explored. Limitations of this study included only evaluating metagenomes from healthy adults in the United States, not examining RNA viruses, or nasopharyngeal microbiomes. As additional metagenomic and metatranscriptomic data sets become available, future studies will be well positioned to apply the framework developed here to investigate the healthy human RNA virome. Future work should aim to further characterize the viral community and ecology of nasal cavities and the healthy human RNA virome. Our results demonstrate the efficacy of implementing machine learning to existing metagenomic data to identify biomarker candidates for environmental sampling. The methods developed here for biomarker identification can be utilized to improve other environmental surveillance applications.

## Data Availability

Metagenomes were downloaded from the HMP (https://hmpdacc.org/hmp/) on NCBI DbGaP (Project Accession phs000228; Table S1). All assemblies were downloaded from IMG (Table S2). The specific contigs comprising the vOTUs identified by the machine learning models are listed in Table S4. Fasta files of the twelve vOTUs identified as biomarker candidates are available on Zenodo (10.5281/zenodo.17178845).

## SUPPLEMENTAL MATERIAL

Supplemental Material msphere.00113-26-s0001.pdf Figures S1 and S2 and supplemental table captions.

Supplemental tables msphere.00113-26-s0002.xlsx Tables S1 to S9.

ASM does not own the copyrights to Supplemental Material that may be linked to, or accessed through, an article. The authors have granted ASM a non-exclusive, world-wide license to publish the Supplemental Material files. Please contact the corresponding author directly for reuse.
