# Supplementary material for: Novel machine learning-based approach to identify viral biomarkers of human respiratory emissions from oral and nasal metagenomes
Source: mSphere. 2026 Apr 13;11(5):e00113-26. doi: 10.1128/msphere.00113-26 (PMC13203971; doi:10.1128/msphere.00113-26)
Supplement: Supplemental Material — Figures S1 and S2 and supplemental table captions. [file msphere.00113-26-s0001.pdf]

Supplementary Information: Novel Machine Learning-based Approach to Identify Viral Biomarkers of Human Respiratory Emissions from Oral and Nasal Metagenomes

Kathryn Langenfeld[1], Peter Arts[2], Abigail Monahan[2], Allyson Criswell[2], Krista R. Wigginton[2,], Melissa B. Duhaime[1,*]*

AUTHOR ADDRESS

[1]Department of Ecology and Evolutionary Biology, University of Michigan, Ann Arbor, MI

[2]Department of Civil and Environmental Engineering, University of Michigan, Ann Arbor, MI

Table S1. Accession numbers and sequencing statistics of metagenomes downloaded from the Human Microbiome Project.

Table S2. Accession numbers, assembly statistics, and viral sorting results of metagenomes used to form vOTUs.

Table S3. Segments of *N. subflava*, *S. salivarius*, and *S. sanguinis* genomes used for read mapping. Segments were selected as the areas including and surrounding amplicons for primers developed by Jung et al. (2018).

Table S4. Taxonomic assignment, functional potential annotation, and coding density of each viral biomarker candidate.

Table S5. Primer sequences for the twelve viral biomarker candidates and the three saliva bacteria biomarkers. The primers for the three saliva bacteria biomarkers were previously developed by Jung et al. 2018.

Table S6. Accuracy of machine learning models for the training and test datasets with all features and ten selected features. Each feature was the relative abundance of a vOTU.

Table S7. Prevalence and mean relative abundance of vOTUs selected by at least one machine learning model in nasal, oral, stool, and skin metagenomes.

Table S8. Results of viral purification experiment with fresh saliva and nasal mucus. The presence of each viral biomarker in the initial, after filtration, after chloroform treatment, and after DNase treatment are provided with the stepwise and overall recovery.

Table S9. Prevalence of viral biomarker candidates, viral biomarker cocktails, saliva bacteria biomarkers, and crAssphage in respiratory (oral and nasal) and non-respiratory (stool and skin) metagenomes.

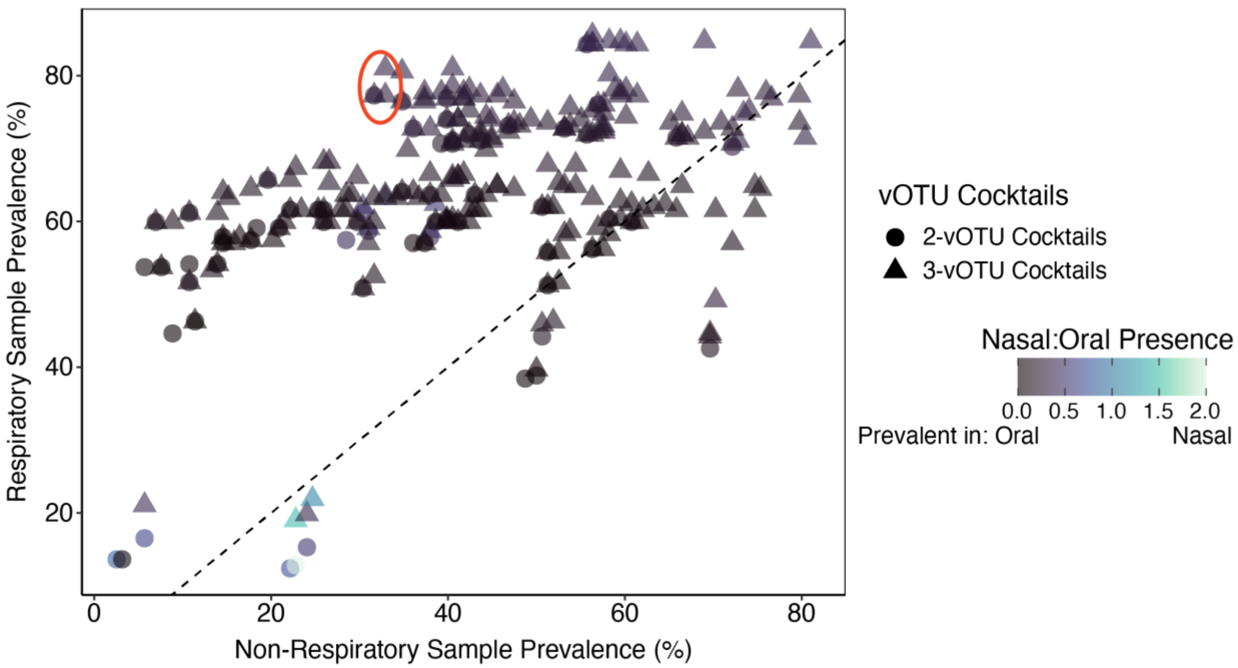

Figure S1. Prevalence results for all of the two-vOTU and three-vOTU combinations for cocktails of viral biomarker candidates. The points indicate the percent of samples with a target present in non-respiratory compared to respiratory samples. A dashed 1:1 line divides which targets are more prevalent in respiratory or non-respiratory samples with points above the line having a higher prevalence in respiratory samples. Points are shaped by the number of vOTUs in each cocktail with two-vOTU cocktails and three-vOTU cocktails as circles and triangles, respectively. Points are colored based on the ratio of the prevalence in nasal compared to oral samples. The red oval highlights the "best" two- and three-vOTU cocktails that are shown in Figure 3 and 4.

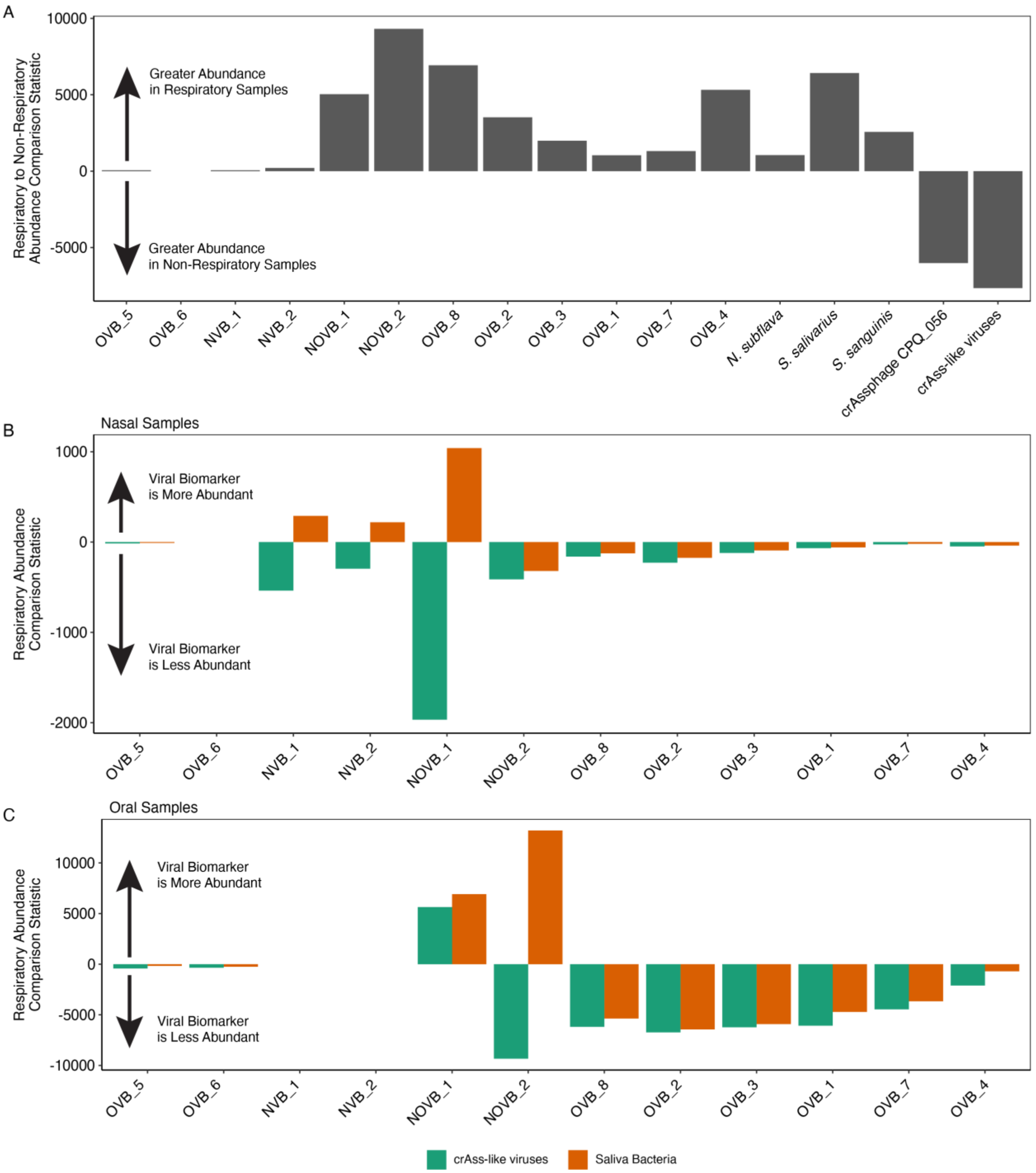

Figure S2. Statistics from Wilcoxon's test comparing biomarker abundances in respiratory samples. Resulting statistics of comparing the relative abundances of each biomarker in respiratory samples compared to non-respiratory samples (A). The statistics from comparing viral biomarker

candidate relative abundances to mean crAss-like viruses relative abundance and mean saliva bacteria relative abundance in nasal (B) and oral (C) samples.
